# *Borrelia burgdorferi* in *Ixodes scapularis* Ticks, Chicago Area

**DOI:** 10.3201/eid1206.060306

**Published:** 2006-06

**Authors:** Dean A. Jobe, Steven D. Lovrich, Jeffrey A. Nelson, Tom C. Velat, Chris Anchor, Tad Koeune, Stephen A. Martin

**Affiliations:** * Gundersen Lutheran Medical Center, La Crosse, Wisconsin, USA;; † North Park University, Chicago, Illinois, USA;; ‡ Forest Preserve District of DuPage County, Wheaton, Illinois, USA;; § Forest Preserve District of Cook County, Elgin, Illinois, USA;; ¶ DuPage County Department of Health, Wheaton, Illinois, USA;; # Cook County Department of Public Health, Oak Park, Illinois, USA

**Keywords:** Lyme disease, Ixodes, Borrelia, Chicago

**To the Editor:** Lyme disease is a multisystem disorder associated with skin, myocardial, musculoskeletal, and central and peripheral nervous system manifestations caused by infection with *Borrelia burgdorferi* sensu lato spirochetes (*1*). In the United States, the illness is caused by transmission of *B. burgdorferi* sensu stricto from the bite of infected *Ixodes scapularis* (deer) ticks found primarily in the northeastern and upper midwestern United States (*2*). *B. burgdorferi*–infected ticks have also been recovered in portions of northern Illinois but have not yet been reported in the Chicago region (*3*). In fact, a previous survey (*4*) of forested areas in the heavily populated regions immediately adjacent to Chicago confirmed a low risk of contracting Lyme disease. Researchers failed to recover deer ticks from 7 sampling sites and recovered only a single *Borrelia* isolate from well-described *B. burgdorferi* sensu stricto rodent reservoirs (mice, voles, chipmunks) captured from 5 sampling sites. A subsequent genetic analysis confirmed the isolate was *B. bissettii* (*5*), a genomic group that is likely nonpathogenic to humans in the United States.

However, the area of the Midwest where Lyme disease is endemic has continued to expand from its origin in northeastern Minnesota and northwestern Wisconsin (*2**,**6*). The Chicago metropolitan region has numerous parks and natural areas that are biologically similar to the known midwestern focus (*7*), and these areas support a large population of white-tailed deer. Moreover, we have seen anecdotal reports of persons with clinical signs and symptoms of Lyme disease and epidemiologic evidence that suggests local acquisition. Two ticks submitted by a resident who had hiked in DuPage County near the east branch of the DuPage River were *I. scapularis*. We therefore began searching for questing ticks in this area and 9 other geographically diverse areas of DuPage County. The 10 sites were flagged by dragging white cotton sheets through the underbrush for 2- to 10-hour intervals during the spring of 2005. We timed these collections on the basis of information that adult deer ticks were questing in Wisconsin. Recovered ticks were placed in sealed vials and transported to the North Park University Laboratory where their identity was confirmed. The midguts were then removed aseptically, and each was placed into a separate vial that contained 2 mL modified Barbour-Stoenner-Kelly medium that could support the growth of small numbers of *B. burgdorferi* sensu stricto (*8*). Cultures were incubated for 6 weeks at 35°C and examined weekly for spirochetes by darkfield microscopy.

Deer ticks were not found at 8 of the DuPage County sample sites. However, 90 adult or nymphal *I. scapularis* were collected from the remaining 2 sites, and spirochetes were recovered from 3 (3%) of the tick midgut cultures. Because of these findings and anecdotal reports of deer ticks in Cook County, we also surveyed 3 sites in Cook County during the fall of 2005. Deer ticks were not found at 2 sites, but 37 adult *I. scapularis* ticks were collected from a site in the southwestern portion of Cook County, and spirochetes were recovered from 2 (5%) cultures.

We first examined the protein profiles to identify the spirochetes. Sodium dodecyl sulfate–polyacrylamide gel electrophoresis analyses of 4 of the 5 isolates (1 isolate lost viability before analysis) showed that they were distinctly different from the *B. bissettii* isolate recovered previously from Cook County (*4*), but the isolates were strikingly similar to *B. burgdorferi* sensu stricto (Figure). We then amplified an intergenic spacer region of the *rrf-rrl* portion of the rRNA from 2 isolates by a previously described method (*9*) and sequenced the amplified products (sequencing by Laragen, Inc., Los Angeles, CA, USA). The sequences were identical to that of *B. burgdorferi* sensu stricto isolate B-31 (*9*).

**Figure:**
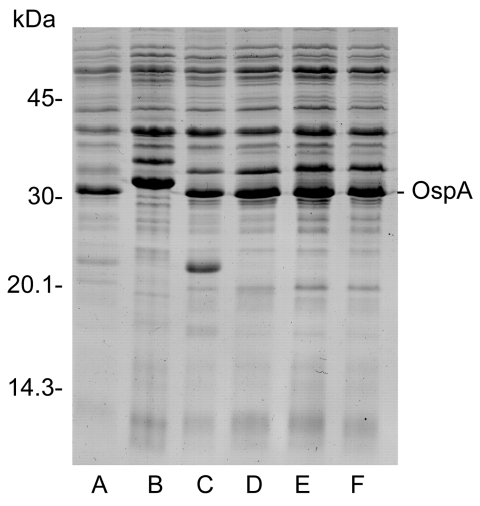
Protein profiles of *Borrelia burgdorferi* sensu stricto (lane A), *B. bissettii* (lane B), and spirochetes from *Ixodes scapularis* ticks collected from DuPage County (lanes C, D) or Cook County (lanes E, F), Illinois.

The results confirmed that *I. scapularis* ticks infected with *B. burgdorferi* sensu stricto were recovered from forested areas surrounding Chicago. Additional studies to define the extent and severity of the risk are necessary, but clinicians and the public should be aware of the possibility of acquiring Lyme disease in the Chicago metropolitan region.
